# Respiratory Muscle Paralysis Associated With Colistin, Polymyxin B, and Muscle Relaxants Drugs: A Case Report

**DOI:** 10.1177/2324709616638362

**Published:** 2016-03-14

**Authors:** Thein Myint, Martin E. Evans, Donna R. Burgess, Richard N. Greenberg

**Affiliations:** 1 University of Kentucky Medical Center, Lexington, KY, USA; 2 University of Kentucky HealthCare, Lexington, KY, USA

**Keywords:** respiratory muscle paralysis, colistin, polymyxin B, muscle relaxant drugs

## Abstract

Polymyxins B and E (colistin) exert a bactericidal effect on the gram-negative bacterial cell wall, causing permeability changes in the cytoplasmic membrane, leading to cell death. Their use was substantially decreased in clinical practice from the 1970s to 2000s due to their significant nephrotoxicity and neurotoxicity compared to the newly introduced antibiotics. The increasing prevalence of multidrug-resistant gram-negative bacteria infections in this century has led to an upsurge in the use of these “older” drugs. Respiratory paralysis caused by neuromuscular blockage associated with the use of polymyxin B and E was reported mostly in literature published in the 1960s to 1970s with a few reports after 2000. In addition, such a reaction might be enhanced by the presence of other classes of drugs. We report a case of polymyxin B and E–induced apnea in a patient receiving “muscle relaxants.”

## Introduction

Polymyxins B (PMB) and E (colistimethate sodium, colistin [CMS]) exert a bactericidal effect on the gram-negative bacterial cell wall, causing permeability changes in the cytoplasmic membrane, leading to cell death. They were introduced into medical practice in the 1950s but their use was substantially decreased from the 1970s to 2000s due to significant nephrotoxicity and neurotoxicity compared to the newly introduced antibiotics. The increasing prevalence of multidrug-resistant gram-negative bacteria infections in this century has led to an upsurge in the use of these “older” drugs. Reports of potentially severe and life-threatening respiratory paralysis caused by neuromuscular blockage associated with their use have been published, mostly in the 1960s to 1970s.^1,2^ Reports also suggested that polymyxin-related neuromuscular blockade might be enhanced by the presence of other drugs with similar toxicities such as neomycin,^3^ anesthetics,^4^ and neuromuscular blocking agents,^5^ including pipecuronium.^6^ We report a case with episodes of both CMS and subsequent PMB-induced apnea in a patient receiving “muscle relaxants.”

## Case Report

**Figure 1.**
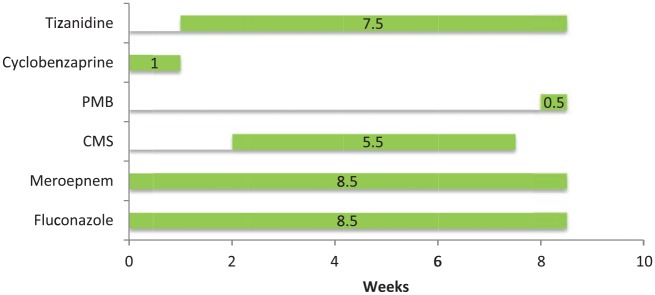
Time Line of Antibiotics and muscle relaxants.

A 57-year-old male with history of diabetes mellitus, atrial fibrillation, and degenerative joint disease underwent a left L4-L5 hemilaminectomy, medial facetectomy, and microdiskectomy to relieve the symptoms of spinal stenosis. The surgery was complicated by a methicillin-sensitive *Staphylococcus aureus* (MSSA) bacteremia. A surface culture of the open surgical site grew MSSA, *Candida albicans*, and multidrug resistant *Pseudomonas aeruginosa* (resistant to levofloxacin; minimum inhibitory concentration [MIC] >16 µg/mL), intermediate susceptibility to cefepime (MIC = 8 µg/mL) and piperacillin/tazobactam (MIC = 32 µg/mL), but sensitive to meropenem (MIC <1 µg/mL). A surveillance culture 1 month prior to this wound infection did not grow a multidrug-resistant *Pseudomonas aeruginosa*. He was discharged to a nursing home to receive intravenous (IV) meropenem 1 g every 8 hours and oral fluconazole 400 mg daily. He was also prescribed the muscle relaxant cyclobenzaprine (Flexeril) 10 mg 3 times a day as needed for muscle spasm. One week later, cyclobenzaprine was changed to tizanidine (Zanaflex) 1 mg at bedtime. Two weeks later, when *Pseudomonas aeruginosa* resistant to meropenem (MIC = 8) was recovered from an open wound with a purulent discharge, IV CMS was started at 3 mg/kg/day divided into 2 doses, and the dose of IV meropenem was increased to 2 g every 8 hours. CMS was chosen rather than an aminoglycoside as his infectious disease physician believed this offered the patient the best chance to receive a possible synergic combination to treat the multiresistant *Pseudomonas*.^7^ After completing the 3 weeks of IV CMS, and 5 weeks of meropenem and fluconazole, he was discharged from the nursing home. His tizanidine dose was increased to 4 mg every 8 hours 2 weeks later. He received 3 additional weeks of IV CMS and IV meropenem at home (Figure 1). During this time he became progressively weak with frequent falls and also became short of breath. During the third week at home, he stopped talking, could not move his arms, and had labored respirations. He was intubated when seen as an emergency and brought to a hospital. His serum electrolytes and renal function tests are shown in Table 1. His serum creatinine had risen from 0.6 mg/dL prior to CMS to 1.1 mg/dL. A creatinine clearance was 110 mL/min at this admission. Computed tomography (CT) showed no intracranial lesions. A CT angiography of the head and neck showed no significant stenosis. He was given tissue plasminogen activator for the treatment of a probable ischemic stroke. Tizanidine and CMS were discontinued and PMB started with a loading dose of 160 mg IV followed by 100 mg every 12 hours. IV meropenem and oral fluconazole were also restarted. He was extubated 1 day after the admission.

**Table 1.** Laboratory Results Before, on the Day of Admission, and on the Third Day of Admission.

Labs	Normal Range	Prior to Starting Colistin	2 Weeks After Colistin	5 Weeks After Colistin	6 Weeks After Colistin (on the Date of Admission)	Third Day of Admission
Potassium	3.7-4.8 mmol/L	4.5	4.5	3.9	3.0	3.5
Magnesium	1.9-2.4 mg/dL	NA	1.5	1.7	0.9	1.5
Calcium	8.9-10.2 mg/dL	8.4	9.2	9.5	9.3	8.8
Corrected calcium	8.9-10.2 mg/dL	10.0	10.7	10.7	10.8	NA
Albumin	3.3-4.6 g/dL	2.0	2.1	2.5	2.1	NA
Creatinine	0.8-1.3 mg/dL	0.62	1.11	1.17	1.09	0.99
White blood cell	3.7-10.3 k/µL	8.0	6.1	9.5	8.7	8.7
C-reactive protein	0-0.9 mg/dL	5.4	2.1	5.4	NA	10.4

Abbreviation: NA, not available.

On the fourth day after admission (and no longer receiving a muscle relaxant), he developed slurred speech, inability to move both his upper extremities, and apnea 10 minutes after completion of an infusion of PMB. He was intubated again. A CT scan of his head did not show changes, and his electroencephalogram was within normal limits. His antibiotics were discontinued (Figure 1). Three days later he was extubated as his right upper extremity weakness had improved. It was noted that his peripheral eosinophil count had increased to 7% after 4 weeks of meropenem and fluconazole and 2 weeks of CMS, and then to 11% 2 weeks later. It was 19% at the time when all antibiotics were stopped. One month later it had fallen to 10%. The patient was not restarted on any antibiotics. The wound was treated with wet to dry dressing changes and debrided 5 months later. The patient remains with a chronic nonhealing wound.

## Discussion

Polymyxins B and E are small basic peptides (molecular weight ~ 1000) and cationic detergents that exert a bactericidal effect on the gram-negative bacterial cell wall, causing permeability changes in the cytoplasmic membrane, leading to cell death.^1,8^ Their use was substantially decreased in clinical practice from the 1970s to 2000s due to their significant nephrotoxicity and neurotoxicity compared to the “newly” introduced antibiotics.^9^ The increasing prevalence of multidrug-resistant gram-negative bacteria infections in this century has led to an upsurge in the use of PMB and CMS.

Respiratory muscle paralysis is a rare but potentially fatal complication of the use of PMB and CMS.^2^ The incidence of CMS associated neurotoxicity reported in the literature prior to 1975 ranged from 7.3% to 27%, with paresthesia constituting most of the reports.^1,10^ Lindesmith et al^2^ described 21 cases of reversible respiratory paralysis in 1968; 15 cases were associated with CMS and 6 cases with PMB therapy. The number of doses associated with episodes of respiratory arrest ranged from a single dose to 45 doses of antibiotics. Onset of paralysis occurred from 1 to 26 hours after a dose of PMB or CMS. Most of those cases were described in patients with renal disease,^2^ suggesting that the risk of polymyxin-associated neuromuscular blockade is increased with impaired renal function.

Other neurotoxic effects include circumoral paresthesia or numbness, tingling or formication of the extremities, generalized pruritus, vertigo, dizziness, and slurring of speech.^1,2,9^ Untreated neurotoxicity associated with PMB or CMS is a precipitating factor for respiratory muscle paralysis and respiratory failure.

The proposed mechanism of CMS neurotoxicity is a noncompetitive myoneuronal presynaptic blockade of acetylcholine release that may be enhanced by hypocalcemia-induced prolongation of depolarization.^9^ Concomitant drug therapies including other neurotoxic drugs (anesthetics, aminoglycosides, and paralytics), corticosteroids, narcotics, and muscle relaxants (which were given to this patient) probably increase the risk of CMS neurotoxicity.^1,9^

After the resurgence in the use of CMS and PMB for multidrug-resistant gram-negative bacteria in this century, there have been several case reports of PMB- or CMS-related respiratory apnea that required intubation.^11-17^ Seven of 9 cases were associated with CMS (Table 2).

**Table 2.** Case Reports Summary of Colistin/Polymyxin-Induced Apnea That Required Intubation, Published After 2010.

No.	Study	Age	Gender	Indication	IV Drug and Dose	Duration of Antibiotic Administration Prior to Onset of Apnea	Duration of Endotracheal Intubation, Outcome	Associated With Underlying Renal Disease	Concomitant Neurotoxic Drugs
1	Wahby et al^11^ (2010)	33	Female	MDR *Acinetobacter baumannii*	170 mg of colistin base activity every 12 h	5 days	5 days, survived	NA	Methylprednisolone, hydromorphone
2	Spapen et al^12^ (2011)	51	Male	New Delhi metallo-β-lactamase-1 *Escherichia coli*	Colistin 3 million units every 8 h	19 days	NA, died	No	Anesthetic agent
3	Wunsch et al,^13^ case 1 (2012)	48	Male	Carbapenem resistant *Klebsiella pneumoniae*	Polymyxin 125 mg every 12 h	1 hour	NA	No	IV amikacin
4	Wunsch et al,^13^ case 2 (2012)	58	Male	MDR *Klebsiella pneumoniae*	Polymyxin B 80 mg every 12 h	2 days	1 day	Yes	NA
					Second test dose of IV polymyxin	2 hours	1 day, survived		
5	Fernandez et al^14^ (2013)	75	Male	MDR *Pseudomonas aeruginosa*	Colistin 3 million international units every 8 h	36 hours	1 day, died	No	NA
6	Wadia and Tran^15^ (2014)	51	Male	*Acinetobacter baumannii*, ESBL *Proetus mirabilis*	Colistin 275 mg q12h	8 days	NA, survived	No	NA
7	Shrestha et al^16^ (2014)	31	Female	Pan resistant *Pseudomonas*	Colistin 200 mg q12h	3 days	1 day, survived	Yes	NA
8	Nigam et al^17^ (2015)	20	Female	MDR *Pseudomonas*	Colistin 1 million units q8h	5 days	No intubation, survived	NA	No
9	Our patient	57	Male	MDR *Pseudomonas aeruginosa*	Colistin 120 mg IV q12h; polymyxin B 100 mg IV q12	6 weeks, and again for only 3 days	3 days twice, survived	No	Tizanidine

Abbreviations: MDR, multidrug-resistant; NA, not available; IV, intravenous.

Our patient received intravenous CMS 3 mg/kg/day divided into 2 doses for 6 weeks. He did not appear to have underlying renal insufficiency prior to admission, and his creatinine clearance was never below 95 mL/min during his hospital stay. His serum calcium was within normal limits. He did not have tetany, arrhythmia, seizure, or involuntary movements although he was slightly hypokalemic and hypomagnesemic. He developed shortness of breath after a prolonged course of IV CMS and apnea after 3 days of PMB. He developed eosinophilia but did not have a rash or organ injury. Stool parasite studies were negative as was serum antibody for *Strongyloides stercoralis*. Eosinophilia associated with CMS or PMB has been reported in 1.6% of patients receiving these drugs.^1^ Our patient’s eosinophilia could have resulted from exposure to one or more of medications that included meropenem, fluconazole, tizanidine, CMS, and PMB.

Neurological diseases such as stroke and seizure were excluded in this patient. He was not receiving any steroids. His respiratory paralysis reversed after stopping PMB and CMS.

The patient received the muscle relaxants cyclobenzaprine (Flexeril) and tizanidine (Zanaflex), which could have potentially increased neuromuscular blockage leading to his first episode of respiratory muscle paralysis, though this interaction has not been reported.^9^ The dose of tizanidine, 4 mg every 8 hours, was also relatively high. He did not receive concurrent curariform muscle relaxants or other neurotoxic drugs (tubocurarine, succinylcholine, gallamine, decamethnium, and sodium citrate), which have been associated with respiratory depression.^1^ The patient’s CMS- and PMB-induced apnea might have been enhanced by the muscle relaxants. His Naranjo adverse drug reaction probability scale of 7 indicates these event were probably an adverse drug reaction.^18^

With an increasing use of IV CMS and PMB to treat multidrug-resistant gram-negative bacteria infections, clinicians must be aware of respiratory muscle paralysis as a rare and potentially fatal side effect of these drugs. Patients receiving these drugs should have regular reviews for drug-drug interactions, as well as renal and neurological assessments.^11^ If the patient develops neurotoxicity, CMS or PMB should be stopped. There is no role for reversal agents such as neostigmine, although calcium infusions and antihistamines have been suggested to have some benefit in reversing the paralysis.^2^ As this reaction can be reversible, continuous renal replacement therapy or hemodialysis seems a reasonable option to help in patients with acute renal failure or with high serum levels of polymyxins.
